# Do bystanders always see more than the players? Exploring Solomon’s paradox through meta-analysis

**DOI:** 10.3389/fpsyg.2023.1181187

**Published:** 2023-08-07

**Authors:** Hongyi Lin, Hong Zheng, Fengyan Wang

**Affiliations:** School of Psychology, Nanjing Normal University, Nanjing, China

**Keywords:** Solomon’s paradox, wise reasoning, wisdom, interpersonal conflict, meta-analysis

## Abstract

Solomon’s paradox is a widespread phenomenon regarding how we think, which asserts that people reason more wisely about other people’s social problems than they do about their own. This means that we are more likely to make rational decisions when decision-making on the behalf of others than for ourselves, which has practical implications in the field of interpersonal conflicts and social dilemmas. However, it remains unclear whether Solomon’s paradox exists across cultures, and the magnitude of its effect size. A meta-analysis was conducted, examining six studies and 20 effect sizes, to gain more insight into this phenomenon, considering the influencing effects of culture, measurement instrument, conflict type, and some other moderating factors. The results showed that Solomon’s paradox does exist in interpersonal conflict (*d* = 0.317; 95% CI = 0.828–0.852). Moderator analysis revealed that measurement instrument and subjects had an impact on the effect of Solomon’s paradox and there was a non-significant effect size of culture and conflict type. Future research should explore the diverse forms of Solomon’s paradox across more diverse cultural contexts (e.g., various countries) to better understand the phenomenon and help people cope with life’s problems more wisely.

## 1. Introduction

King Solomon, the third leader of the Jewish Kingdom, and was renowned for his ability to advise others soundly, and is often portrayed as a paragon of wisdom, famed throughout his kingdom for his sage judgment. There was even a popular saying: If you have any questions, ask Solomon, and he will tell you what to do. Many people traveled far to seek his counsel. However, he also made many wrong and even foolish decisions when dealing with his problems. Eventually his extravagance and poor choices in his later years led to the downfall of his dynasty. This phenomenon, of being a wise advisor to others but a poor decision-maker in personal issues, was named Solomon’s paradox by Grossmann and Kross (2014), with the concept also existing in Chinese proverb as “当局者迷，旁观者清” (The bystander will always see more than the player) (Liu et al., 2013).

Solomon’s paradox defines the human trait of being better able to reason wisely about the conflicts or dilemmas of others than those we face personally, and represents a fundamental and widespread social cognitive bias. Grossmann and Kross (2014) experimentally tested the existence of Solomon’s paradox for the first time. They randomly assigned both groups to situations of self-conflict or other’s conflict, and asked participants to complete a wise reasoning scale after recalling details about the conflict. The results showed that individuals in other’s conflict showed higher levels of wise reasoning (Grossmann and Kross, 2014). Now, Solomon’s paradox has already received much attention in many studies (Huynh et al., 2017; Xu et al., 2022).

Grossmann and Kross (2014) found that people are less likely to adopt multiple wisdom-related strategies when reasoning about their own personal issues than when considering others’ conflicts. This has been seen in both younger and older adults, providing evidence for Solomon’s paradox (Grossmann and Kross, 2014). Grossmann et al. (2010, 2013) regarded wisdom (reasoning) as practical reasoning related to wisdom used by individuals to cope with many challenges in social life, while wise reasoning consists of the common features of dialectical thinking, intellectual humility, compromise-seeking, and prosocial tendencies to promote the common good, all of which require one to go beyond egotism and consider and reason about an overall situation holistically (Staudinger and Glück, 2011). Studies have shown that, when people are thinking about important life problems, they tend to focus on the specific details of their own self in their own life experiences, which makes it difficult for them to broaden their perspectives and is not conducive to reasoning (Ayduk and Kross, 2010; Grossmann and Kross, 2010). Reflecting on personal experiences from the perspective of a bystander or through self-abstraction, however, individuals are able to adopt more abstract high-level explanations to represent and avoid egocentric perspectives (Trope and Liberman, 2010; Kross and Ayduk, 2011). This method of self-distance has also been observed in an intervention study in which participants were trained to reflect on daily conflicts in the third person, and results showed that the participants were able to effectively improve their wise reasoning in this manner, as compared to when they thought about their problems in the first person (Grossmann et al., 2021).

The existing research indicates that people’s perceptions of themselves are often less accurate than their perceptions of other people (Nisbett and Ross, 1980; Pronin, 2008), another detail with several practical implications. When encountering interpersonal conflict, people thinking blindly from their own position may not be able to consider the larger situation objectively and comprehensively, leading to the aggravation of the conflict. However, Solomon’s paradox impacts many more social dilemmas than simply interpersonal conflict (e.g., management, intergroup negotiations, even between countries). For example, a third-party individual in an advisory role attempting to mediate a conflict while considering the situation from a position of their own self-interest would lead to unfair outcomes, as their decisions or advice would be affected by their own personal stakes (Grossmann and Kross, 2014).

Research into Solomon’s paradox has had important practical value, shedding light on how to better navigate the fields of social dilemmas and interpersonal conflicts. However, there are still some shortcomings in the existing research, and the details of the effects of Solomon’s paradox are still controversial. Findings on the effect size of Solomon’s paradox have varied greatly (Grossmann and Kross, 2014; Xu et al., 2022), yet even this basic but important detail is not noted in some sample surveys (Ren, 2021). Existing studies of Solomon’s paradox have taken the form of single empirical investigations, conducted in their respective cultural contexts and lacking generalizability. Furthermore, to our knowledge, there has not yet been any meta-analysis done to examine the effects of Solomon’s paradox. Thus, it would be beneficial to carry out a systematic review integrating the available effect sizes. Therefore, the current review used meta-analysis to systematically integrate the relevant literature to verify the existence of Solomon’s paradox, define its effect size, and compare the moderating effects of variables such as different cultures, different subjects, and measurement tools. We hope to explore the Solomon’s paradox more clearly through this review to help people cope with life’s problems more wisely.

## 2. Method

### 2.1. Inclusion criteria and search strategy

**Figure 1:**
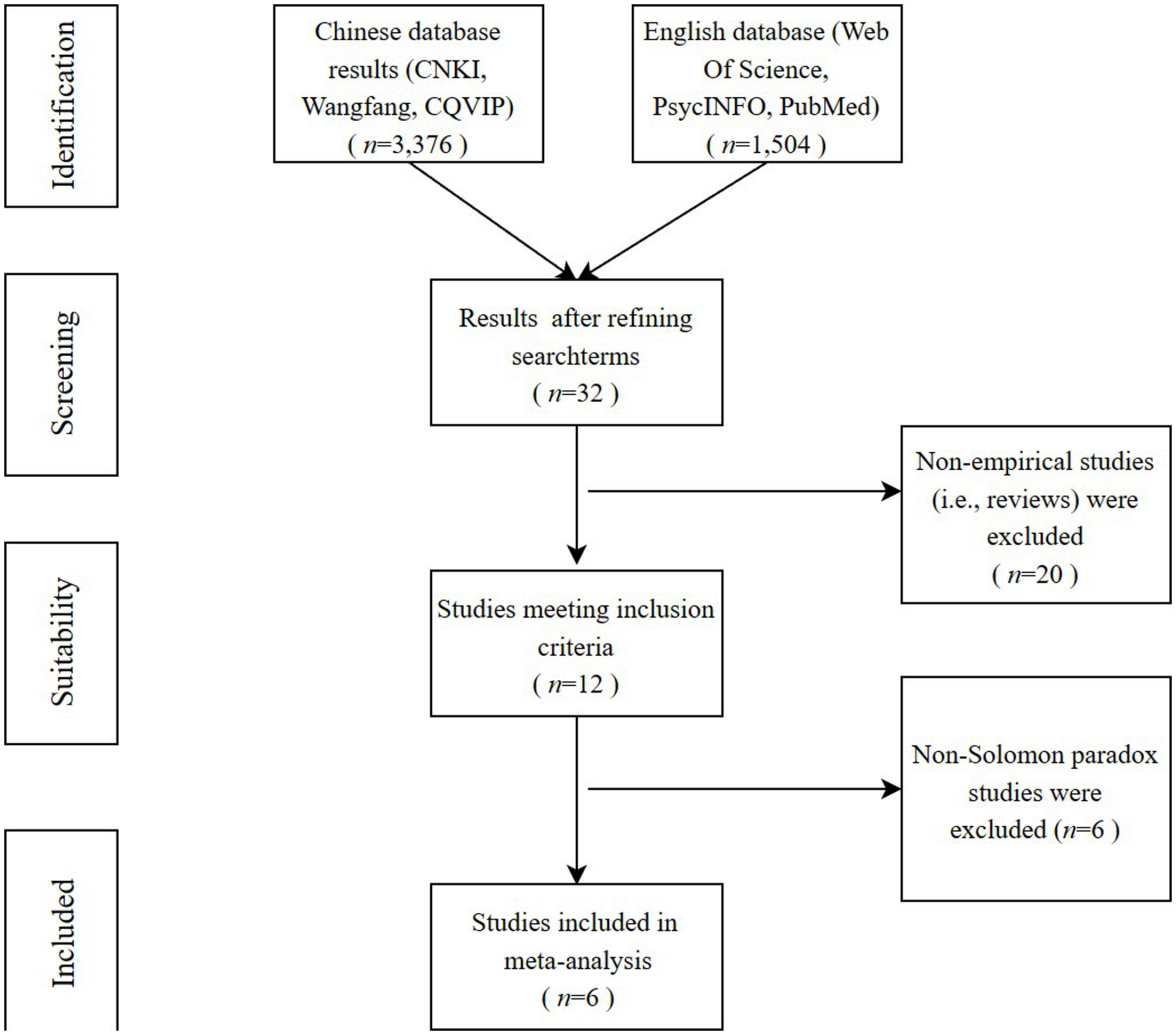
Workflow chart of the selected study review and evaluation process.

The current study began with a comprehensive search for relevant literature published in Chinese or English. The CNKI, Wanfang Data, and CQVIP databases were used to find journal articles and dissertations written in Chinese, while PsycINFO, PubMed, and Web of Science Core Collection were searched for journal articles and dissertations written in English. The initial database search looked for abstracts only. Meanwhile, the researchers also combed the reviews and references of the relevant articles dated until January, 2023. Keywords used in the search of the Chinese databases included “智慧” and “冲突,” while the English-language search used “wisdom*” and “conflict *” as keywords. A total of 4,840 results were returned from the searches. These 4,840 studies were subsequently screened thoroughly by three researchers working together to determine whether they fit the following criteria, as outlined in Figure 1: (1) they must have been empirical studies with first-hand information; (2) the research theme was wisdom psychology; (3) data for experimental versus control groups were reported clearly or could be calculated; and (4) the article was not a repeat of another publication which was already included in the meta-analysis. Ultimately, six studies ultimately met the inclusion criteria to be used in this meta-analysis, and were imported into EndNote X7.

### 2.2. Data extraction and encoding

We assessed each study to determine if it examined whether context, that is, self-conflict versus others-conflict, affect one’s level of wise reasoning. Other possible moderating factors, such as participant demographics methodological differences, or publication characteristics, were also taken into account.

The following data were extracted for all included studies: first author, study area, cultural region, measurement tools, *M* and *SD* of experimental and control groups, sample size, and demographic characteristics of participants, including their age and gender. Two psychology postgraduates encoded the selected articles and dissertations individually. Coding disagreements were discussed with the intervention of a third person to determine the final results. The included studies and their associated characteristics can be found in the Supplementary material.

### 2.3. Data processing

Comprehensive Meta-Analysis Version 3.0 was used to perform the meta-analysis of the differences in effect size of wise reasoning between the self-and other-conditions. Statistical heterogeneity was examined using *I*^2^ and with a *p* value for *Q* statistics. *I*^2^ values represent the degree of heterogeneity, indicating low (≤ 25%), medium (50%) and high (≥ 75%) heterogeneity (Higgins and Thompson, 2002). To perform the moderator analyses, the categorical variables were transformed into dummy variables, and continuous variables were centered on their average. As this analysis comprised only a few studies, publication bias was not investigated (Liang et al., 2022).

The methodological quality of the studies included was assessed using an 11-item checklist, as recommended by the Agency for Healthcare Research and Quality (AHRQ). An item would be given a score of “0” if the answer was “NO” or “UNCLEAR”; if the answer was “YES’,” then the item was given a score of “1”. Two reviewers independently evaluated the methodological quality of each included study, using the 11 AHRQ criteria and calculating an overall score for each to determine the article quality. Article quality was rated as follows: low quality = 0 to 3; moderate quality = 4 to 7; high quality = 8 to 11 (Hu et al., 2015). Discrepancies regarding quality ratings were discussed until consensus was reached, with a third researcher making the final decision if agreement could not be reached.

Cohen’s *d* was used to calculate the effect sizes of each outcome measure using the formula developed by Lipsey and Wilson (2001). Effect sizes were calculated for the self-condition and other-condition measurements. Subsequently, the self-condition effect sizes were subtracted from the other-condition effect sizes. If any measures for calculating the Cohen’s *d* could not be coded directly from the study, they were converted. Cohen (1992) suggests the following interpretation of effect sizes: *d* = 0.20 is a small effect, *d* = 0.50 is a medium effect, and *d* = 0.80 is a large effect.

## 3. Results

### 3.1. Characteristics of the selected studies

A total of six articles, including 20 independent samples, were retrieved for this meta-analysis, comprising 835 participants (other-condition group *N*_other_ = 2,377, self-condition group *N*_self_ = 2,401). Regarding study region, eight independent samples were from United States (Grossmann and Kross, 2014; Huynh et al., 2017; Wei and Wang, 2021; Xu et al., 2022) and one from Britain (Huynh et al., 2017). The remaining samples were from Asia, all of which were from China (Xu, 2019; Ren, 2021; Wei and Wang, 2021; Xu et al., 2022). Of the total research group, 50% consisted of college students, and the average age of participants in most of the studies (95%) was under 40 years, with the only exception being one survey of elderly participants (Grossmann and Kross, 2014). Regarding the detailed measure components, two research instruments were used to assess the reported differences in wise reasoning across the two individual conditions: the Situated Wise Reasoning Scale (SWIS) and a procedure for wise-reasoning questions. The SWIS is a five-point self-rated scale consisting of 21 items and five dimensions (Brienza et al., 2018). Meanwhile, the procedure to assess individuals’ wise reasoning was similar to the SWIS, but some of the items (i.e., responses to changed items) were scored by condition-blind judges who counted the number of outcomes listed by the participants (Grossmann and Kross, 2014; Kross et al., 2014).

### 3.2. Quality assessment

Table 1 depicts the quality assessment of the included studies. All studies had clear recruitment criteria and used scientific measurement tools. All studies fulfilled at least five of the 11 criteria items, and no studies had a low quality rating, meaning that the overall resulting quality of the studies was high, with a mean score of 7.55.

**Table 1:** Methodological quality assessment of included studies.

Study	Assessment items											
	Q1	Q2	Q3	Q4	Q5	Q6	Q7	Q8	Q9	Q10	Q11	Total
Xuwentao2019-1	1	1	0	1	1	0	1	0	1	1	0	7
Xuwentao2019-2	1	1	0	1	1	0	0	1	0	0	0	5
Weixindong2021-1	1	1	0	1	1	1	1	1	1	1	0	9
Weixindong2021-2	1	1	0	1	1	1	1	1	1	1	0	9
Weixindong2021-3	1	1	0	1	1	0	1	1	1	1	0	8
Weixindong2021-4	1	1	0	1	1	0	1	1	1	1	0	8
Weixindong2021-5	1	1	0	1	1	0	1	1	1	1	0	8
Weixindong2021-6	1	1	0	1	1	0	1	1	1	1	0	8
Weixindong2021-7	1	1	0	1	1	0	0	1	0	0	0	5
Weixindong2021-8	1	1	0	1	1	0	0	1	0	0	0	5
Renguoqing2021-1	1	1	0	1	1	0	1	0	1	1	0	7
Renguoqing2021-2	1	1	0	1	1	0	1	0	1	1	0	7
Renguoqing2021-3	1	1	0	1	1	0	1	0	1	1	0	7
Xuwentao2022	1	1	1	1	1	0	1	0	0	0	0	5
Huynh2017-1	1	1	1	1	1	1	1	1	1	1	0	10
Huynh2017-2	1	1	1	1	1	1	1	1	1	1	0	10
Grossmann2014-1	1	1	1	1	1	1	1	1	1	1	0	10
Grossmann2014-2	1	1	1	1	1	1	1	1	0	1	0	9
Grossmann2014-3	1	1	1	1	1	1	1	1	1	1	0	10
Grossmann2014-4	1	1	1	1	1	1	1	1	1	1	0	10

0, NO or UNCLEAR; 1, YES; Q1, Define the information source; Q2, List inclusion and exclusion criteria for exposed and unexposed subjects (i.e., cases and controls) or refer to previous publications; Q3, Indicate time period used for identifying patients; Q4, Indicate whether subjects were consecutive if not population-based; Q5, Indicate whether evaluators of subjective components of the study were blinded to other aspects of participants’ status; Q6, Describe any assessments undertaken for quality assurance purposes; Q7, Explain any patient exclusions from analysis; Q8, Describe how confounding was assessed and/or controlled; Q9, If applicable, explain how missing data were handled in the analysis; Q10, Summarize patient response rates and completeness of data collection; Q11, Clarify what follow-up, if any, was expected and the percentage of patients for which incomplete or follow-up data was obtained.

### 3.3. Sensitivity analysis

The results of the sensitivity analysis showed that the effect size did not fluctuate considerably from the original estimates after excluding any one sample (0.280 ~ 0.334), indicating that the final estimated results of the meta-analysis were stable.

### 3.4. Effect sizes and heterogeneity test

**Figure 2:**
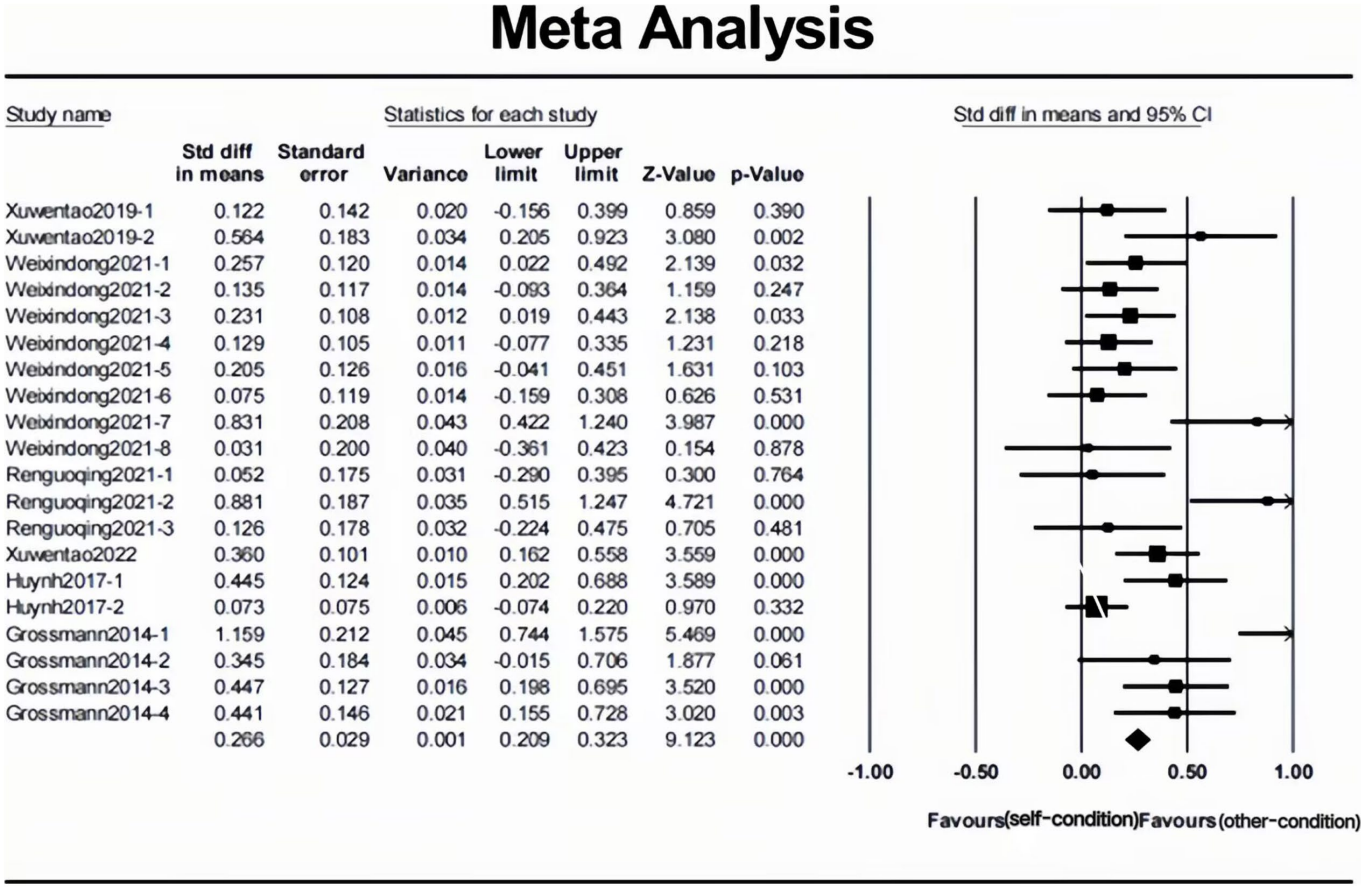
Forest plot of the 20 independent samples included in the meta-analysis.

The heterogeneity test found that the effect size of the Q test was significant, and the *I*^2^ value was greater than 50% (Higgins and Thompson, 2002), indicating that the effect sizes of this study were heterogeneous and may be affected by the moderating variables, meaning that people are indeed wiser when reasoning about others’ problems [*d* = 0.317, 95% CI (0.209, 0.424), *p* < 0.001; see Table 2 and Figure 2]. Although the Cohen’s *d* could be categorized as small (Cohen, 1992), this means that, as expected, Solomon’s paradox does exist in interpersonal conflict.

**Table 2:** Effect size and heterogeneity test results of differences between self-and other-condition.

	*k*	*N*	Effect size and 95% CI			Heterogeneity test	
			Cohen’s *d*	Lower	Upper	*I* ^2^(%)	*p*
Overall effect	20	67,578	0.317	0.828	0.852	69.488	< 0.001

*k*, number of independent studies.

### 3.5. Test of moderating effects

First, meta-regression analysis was used to analyze the moderating effects of gender (percentage of males) and age (average age) on Solomon’s paradox. The results showed that the moderating effect of gender was not significant (*b* = −0.915, *p* = 0.093), and the moderating effect of age was also not significant (*b* = −0.002, *p* = 0.772). The results of the subgroup analysis showed that: (1) the effect size in Western culture was larger than in Eastern culture, but the moderating effects were not significant (Cohen’s *d* = 0.400, 0.257, *p* = 0.234); (2) in the wise reasoning dimension, the effect size of “considering other people’s perspectives” was larger, but there were no significant differences overall; (3) there were significant differences of the research tools used, with the effect size of the Wise-Reasoning procedural questions larger than that of the SWIS (Cohen’s *d* = 0.025, 0.060, *p* < 0.001); (4) the effect size was smaller among working adults than among college students (Cohen’s *d* = 0.432, 0.209, *p* < 0.05); (5) the effect size was larger for general social relationships than for intimate relationships, but there was no significant difference in the type of interpersonal conflict (Cohen’s *d* = 0.473, 0.296, *p* = 0.298); and (6) there were significant differences between sample sizes, with smaller sample sizes resulting in larger effect sizes for Solomon’s paradox (Cohen’s *d* = 0.475, 0.222, *p* < 0.05). See Table 3 for details.

**Table 3:** Effects of moderator analyses of differences between self-and other-condition.

Moderator variables	Heterogeneity test			Subgroup	*k*	Cohen’s *d*	95% CI	
	*Q_B_*	*df*	*p*				Lower	Upper
Cultural region	1.418	1	0.234	Eastern culture	12	0.257	0.129	0.406
				Western culture	8	0.400	0.233	0.593
Wise reasoning dimension	3.281	4	0.512	Considering other people’s perspectives	9	0.354	0.201	0.508
				Intellectual humility	9	0.239	0.086	0.392
				Search for compromise	9	0.236	0.084	0.389
				Outsider’s perspective	5	0.134	−0.063	0.331
		2		Recognition of change	9	0.223	0.069	0.376
Measurement instrument	11.868	1	0.001	SWIS	15	0.225	0.125	0.325
				Wise-reasoning questions	5	0.610	0.415	0.805
Subjects	4.357	1	0.037	College students	10	0.432	0.275	0.590
				Adults (working)	9	0.209	0.070	0.348
Conflict type	1.084	1	0.298	General social conflict	3	0.473	0.182	0.409
				Intimate relationship conflict	17	0.296	0.159	0.787
Sample size	5.826	1	0.016	< 100	9	0.475	0.310	0.640
				> 100	11	0.222	0.100	0.344

SWIS, Items Situated Wise Reasoning Scale.

## 4. Discussion

This meta-analysis (comprising six studies and 20 effect sizes) examined whether Solomon’s paradox exists and its effect size. It also took into account possible moderating variables in terms of participant, methodological, and publication characteristics. Our results show that one’s ability to reason wisely about conflicts or dilemmas that they are involved in is lower than when considering conflicts or dilemmas of others, with a small to moderate positive effect (*d* = 0.317). This finding adds strong evidence that Solomon’s paradox is true, while also affording us a better understanding through which we can improve wise reasoning abilities in interpersonal conflict. However, reference to existing studies (Liang et al., 2022), publication bias was not investigated due to fewer than 10 studies were included in analysis. Therefore, we call on more researchers in different fields to carry out diversified empirical studies in the future to explore Solomon’s paradox.

### 4.1. Gender and age

The relationship between age, gender, and wisdom is crucial in wisdom psychology (Ardelt et al., 2018; Wang and Wang, 2018; Xiong and Wang, 2021). In terms of gender, some researchers have noted that men and women have different relative strengths in wisdom (Treichler et al., 2022), with women scoring higher in compassion-related domains and on items about self-reflection. Furthermore, when experiencing interpersonal conflict, women have been shown to be more likely than men to consider the situation from the other’s perspective (i.e., perspective-taking), and were more accurate in recognizing the limitations of their own knowledge, experiences, and abilities (Grossmann et al., 2012; Booker and Dunsmore, 2016). Rather than shelving or intensifying conflicts, women have been shown to be more inclined than men to solve conflicts by integrating opinions from various parties or seeking compromise (Huynh et al., 2017). Thus, it seems that women are less affected by Solomon’s paradox than men when it comes to interpersonal conflict. In terms of age, previous studies have found that wisdom might increase with age for individuals with the opportunity and motivation to pursue its development (Ardelt, 2010). Thus, it seems that age may also have a moderating effect on Solomon’s paradox. However, no moderating effect of gender and age was found in our meta-analysis. Possible explanations for this could be the lack of existing studies on Solomon’s paradox, as well as the independent samples included in this meta-analysis, four of which reporting only demographic information for their total sample, which was not conducive to refined analysis. In addition, most of the included studies were college students; this means that the age distribution of subjects was relatively concentrated, and furthermore, in college, due to students’ lack of social energy, it might be difficult to note gender differences in wise reasoning. However, we did find significant differences in the effect of Solomon’s paradox between college students and adults (working) in the subgroup analysis. It could be that, compared to college students, working adults or those starting a family are more mentally mature and thus more likely to face more frequent interpersonal conflicts, and as such, their thinking and wise reasoning may grow through this increased presence of difficulties and adversity in their lives (Dorfman et al., 2022). This could then lead them to likely be more considerate of others and to be better able to grasp overall situations rather than simply considering themselves in conflicts, thus, Solomon’s paradox having a lesser impact on them.

### 4.2. Culture

It is notable that no significant difference in effect was found between Western and Eastern cultures, as differences in ways of thought between the two cultures will often affect the reasoning process in conflict (Buchtel and Norenzayan, 2009). Individuals in Chinese culture emphasize the interdependent self, which has an impact on Chinese thinking (Yama and Zakaria, 2019). The self-construct manifests itself as a tendency to consider others in interpersonal communication, and compared with those with independent self-constructs, individuals with interdependent self-constructs are more willing to cooperate with others and adopt others’ views. Furthermore, the independent self is more self-centered, while the interdependent self is more considerate of others (Imamoglu, 2003; Ren, 2021). Additionally, Eastern culture emphasizes collectivism and attaches greater importance to the relationship and links between people. When facing conflict between one’s self and others, it can be difficult for the individual to pull themselves away and think rationally about the conflict situation from the perspective of a third party. Therefore, we expected the effect size of Solomon’s paradox to be lower in Eastern culture than in Western culture. Our meta-analysis did not support this expectation, however. A reason for the similarity between the two could be the lack of overall cultural representation in the included samples. Cultural psychology studies on the psychological and behavioral changes of Chinese people have found that the wealth of resources brought by social development reduces the individuals’ dependence on those around them, and is thus more conducive to the pursuit of personal goals. In addition to the influence of Western individualistic culture, the degree of individualism and the independent self in Chinese culture has gradually become enhanced, while the culture of collectivism and its corresponding values have been steadily declining (Cai et al., 2020). To verify this conjecture, we further differentiated Chinese participants into “independent self” and “interdependent self” for comparison (According to the original author’s own manipulation), and found significant differences between the two groups (*d*_independent self_ = 0.450, *d*_interdependent self_ = 0.179, *p* < 0.05). This result suggests that Solomon’s paradox may be in a “compromise state” between existence and non-existence within current Chinese culture (Wei and Wang, 2021). Although China has a long history of collectivism, under the influence of Western culture, the degree of individualism and independent self in Chinese culture has gradually increased, while the collectivist culture and its corresponding values have decreased (Huang et al., 2018). This verifies the intercultural nature of Solomon’s paradox, to some extent. However, as this meta-analysis included only samples from China, Canada, and the United States, information regarding the cultural interpretations of Solomon’s paradox were still insufficient. We hope that future research will explore Solomon’s paradox in more cultural regions, so as to better form cross-cultural conclusions. Understanding this would will us further resolve interpersonal (and even international) conflict and improve the well-being of all of humanity.

### 4.3. Instrument of measurement and dimensions

Rigorous scientific measurement procedures as well as reliable and valid measurement tools are important factors for the trustworthiness of research results. This meta-analysis found significant differences between measurement instruments for Solomon’s paradox. Compared to the simple self-rating scale, the measurement procedure with observer evaluation had a higher effect size (up to 0.61). This is a particularly interesting result as it can be seen as the Solomon’s paradox of Solomon’s paradox studies. Although the SWIS uses the reconstruction of conflict events to explore respondents’ wise reasoning levels in the face of interpersonal conflict, which has higher ecological validity, it seems, however, that the practice of asking subjects to respond to items using a self-rating scale inevitably traps them in the first-person dilemma. Therefore, by adding observer evaluation (i.e., a third-person perspective), the wise reasoning procedure may more effectively avoid the limitation caused by individuals always evaluating themselves in various situations – which is in fact benefitting from the understanding of Solomon’s paradox. This result also has important reference value for broader psychological measurements. Self-rating scales are often more convenient and efficient, and are more conducive to clinical use and application. However, when measuring key psychological qualities of individuals, it is better to combine evaluation indicators of third-party observers to avoid Solomon’s paradox in the measurement field, to avoid the respondent being influenced by biases such as self-enhancement or accurate self-awareness. “Considering other people’s perspectives” had the highest effect size in terms of measuring the subdimensions of the instrument. When resolving conflicts with others, individuals can think more flexibly when they take the perspective of both sides of a conflict, and switch sides from time to time (e.g., in conflict mediation). When dealing with their own conflicts, as an involved party, it is often not easy for an individual to ignore their own position in the situation to deal with the conflict impartially, especially if it is accompanied by high emotional intensity. An intervention study on wise reasoning also showed that participants reflecting in the third person showed a significant increase in wise reasoning about interpersonal challenges, and these effects were particularly pronounced for intellectual humility and social-cognitive aspects of wise reasoning (i.e., taking other people’s perspectives; Grossmann et al., 2021). However, the results of the current meta-analysis indicate that the overall differences between various dimensions of wise reasoning were not significant, and that more studies are needed in the future to better understand and provide more targeted training for wise reasoning.

### 4.4. Conflict type

In this meta-analysis, conflict type in the included literature was coded as either intimate relationship conflict or general social relationship conflict, both of which were reflected primarily through the difference in interpersonal distance. From the perspective of construal level theory, social distance affects people’s thoughts and behavior (Grossmann and Kross, 2014). The results of this meta-analysis showed that the effect size of conflict in close relationships was smaller than that of general social relationships, but that there was no difference between the conflict types. An increase in social distance has been shown to help improve one’s reasoning ability overall (Förster et al., 2008). Thus, social relationships (e.g., with friends) are more likely to be affected by Solomon’s paradox than intimate conflicts (e.g., with partners or parents). However, the meta-analysis results also showed that there was no significant difference between them in terms of the effect of Solomon’s paradox. A possible explanation for this result could be that although individuals were asked to recall intimate relationship conflicts, there are still specific types of conflicts within this category, and differences in content and degree could affect the study results. The specific content of interpersonal conflict should be further delineated and clarified in future research. Of course, Solomon’s paradox exists not only between people, but also between an individual and a country, or even between countries and other countries. For example, Kross and Grossmann (2012) looked at the career prospects for the unemployed during an economic recession and the results also confirmed the Solomon’s paradox: participants in the distanced group were significantly more likely to recognize the limits of their knowledge, and recognize that the future was likely to change. Thus, the application of Solomon’s paradox across various fields must also be further explored in future studies.

## 5. Limitations

One major limitation of the current meta-analysis is the low number of studies included (*N* = 6). A limited number of studies can jeopardize the validity of statistical conclusions and publication bias, as the number of studies may render insufficient statistical power for detection of an effect. Furthermore, the limited number of studies included in this review also made it impossible to investigate certain moderators (e.g., level of education). Another limitation of the present study is that we focused only on Solomon’s paradox in an interpersonal conflict context. As mentioned above, Solomon’s paradox exists across a wide range of social dilemmas, not only between people. Future research should be more targeted and comprehensive. Furthermore, we hope to see more studies exploring Solomon’s paradox in different cultural contexts in the future, as the limited understanding we have now does suggest that this asymmetry in thinking and decision-making does appear to affect people cross-culturally, existing across people, groups, and countries.

## 6. Conclusion

This meta-analysis of existing literature shows that Solomon’s paradox does not only exist, but it also exists across cultures. The results of our analysis show that the ability of an individual to reason wisely about conflicts or dilemmas in which they themselves are involved is lower than when considering the situations of others. The type of subject and measurement tool were both shown to influence the effect size of Solomon’s paradox, however further studies are needed to explore the effects of this phenomenon in terms of culture, measurement instrument dimension, and type of conflict.

## Author contributions

HL: data collection, analysis and interpretation of results, and draft manuscript preparation. HZ: analysis and interpretation of results, draft manuscript preparation, and enrich the content of the article with processing and polishing. FW: design the article framework, provide constructive comments for the writing of the article. All authors contributed to the article and approved the submitted version.

## Funding

This research was supported by the National Natural Science Foundation of China (Grant No. 31971014).

## Conflict of interest

The authors declare that the research was conducted in the absence of any commercial or financial relationships that could be construed as a potential conflict of interest.

## Publisher’s note

All claims expressed in this article are solely those of the authors and do not necessarily represent those of their affiliated organizations, or those of the publisher, the editors and the reviewers. Any product that may be evaluated in this article, or claim that may be made by its manufacturer, is not guaranteed or endorsed by the publisher.
